# Differential Expression of *Meis2*, *Mab21l2* and *Tbx3* during Limb Development Associated with Diversification of Limb Morphology in Mammals

**DOI:** 10.1371/journal.pone.0106100

**Published:** 2014-08-28

**Authors:** Mengyao Dai, Yao Wang, Lu Fang, David M. Irwin, Tengteng Zhu, Junpeng Zhang, Shuyi Zhang, Zhe Wang

**Affiliations:** 1 Institute of Molecular Ecology and Evolution, East China Normal University, Shanghai, China; 2 Department of Laboratory Medicine and Pathobiology, University of Toronto, Toronto, Canada; Laboratoire de Biologie du Développement de Villefranche-sur-Mer, France

## Abstract

Bats are the only mammals capable of self-powered flight using wings. Differing from mouse or human limbs, four elongated digits within a broad wing membrane support the bat wing, and the foot of the bat has evolved a long calcar that spread the interfemoral membrane. Our recent mRNA sequencing (mRNA-Seq) study found unique expression patterns for genes at the 5′ end of the *Hoxd* gene cluster and for *Tbx3* that are associated with digit elongation and wing membrane growth in bats. In this study, we focused on two additional genes, *Meis2* and *Mab21l2*, identified from the mRNA-Seq data. Using whole-mount in situ hybridization (WISH) we validated the mRNA-Seq results for differences in the expression patterns of *Meis2* and *Mab21l2* between bat and mouse limbs, and further characterize the timing and location of the expression of these two genes. These analyses suggest that *Meis2* may function in wing membrane growth and *Mab21l2* may have a role in AP and DV axial patterning. In addition, we found that *Tbx3* is uniquely expressed in the unique calcar structure found in the bat hindlimb, suggesting a role for this gene in calcar growth and elongation. Moreover, analysis of the coding sequences for *Meis2*, *Mab21l2* and *Tbx3* showed that *Meis2* and *Mab21l2* have high sequence identity, consistent with the functions of genes being conserved, but that *Tbx3* showed accelerated evolution in bats. However, evidence for positive selection in *Tbx3* was not found, which would suggest that the function of this gene has not been changed. Together, our findings support the hypothesis that the modulation of the spatiotemporal expression patterns of multiple functional conserved genes control limb morphology and drive morphological change in the diversification of mammalian limbs.

## Introduction

**Figure 1:**
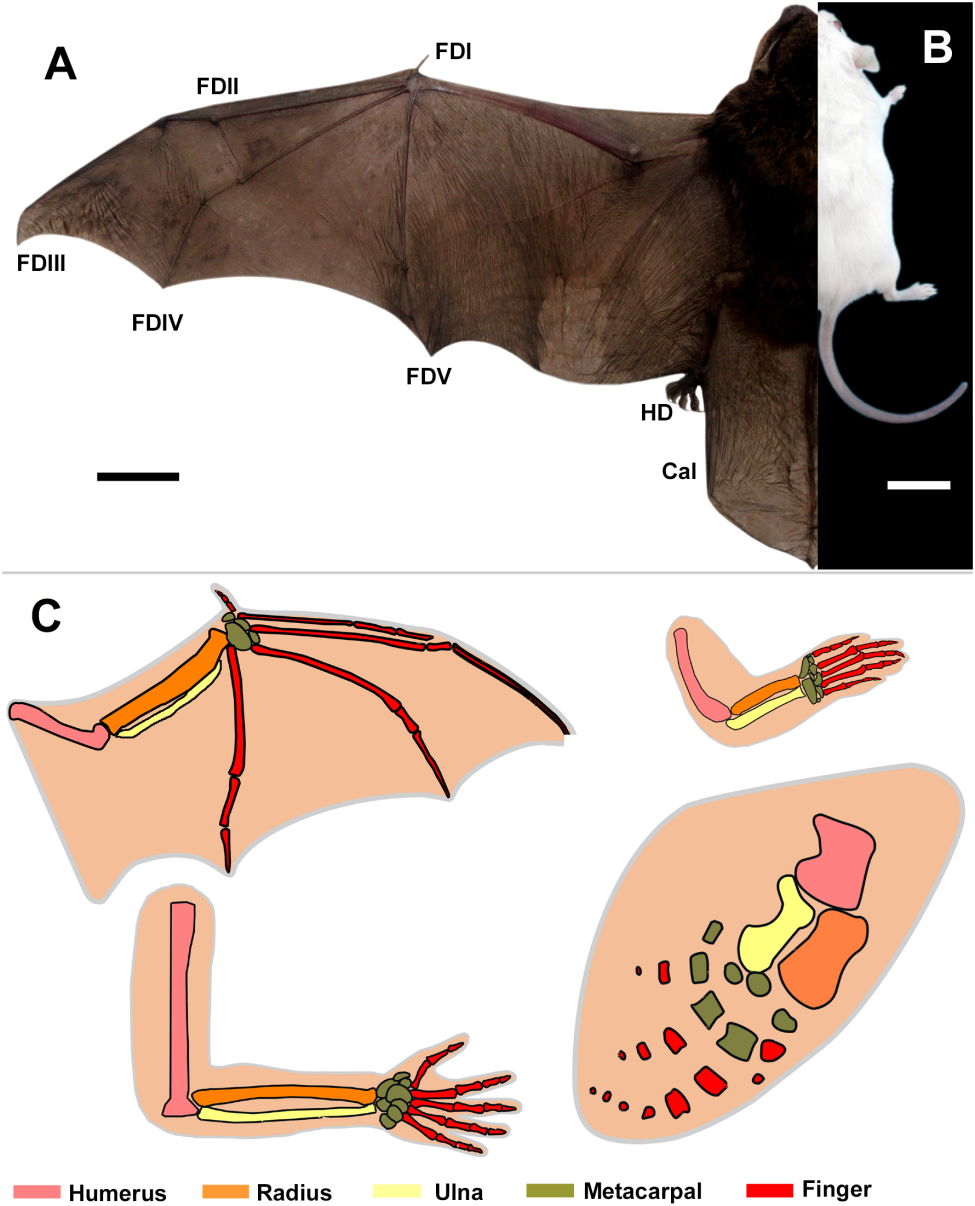
Morphological comparison and schematic diagram of mammalian limbs. (A, B) Morphology of adult mouse (*M. musculus*) and bat (*M. schreibersii*) limbs. FDI, FDII, FDIII, FDIV and FDV: forelimb digits I, II, III, IV and V; HD: Hindlimb digits; Cal: calcar. (C) Schematic diagram of bat, mouse, human and whale limbs. The various colors indicate bones of various groups Scale bars, 2 cm in (A–B).

Bats (order Chiroptera) occupy more than 20% of extant mammalian diversity [1], and are the only living mammals to possess the unique capacity for true self-powered flight. A series of morphological structural changes are associated with bat flight. Bat wings (patagium) are membranous, elastic and thin and supported by skeletal elements in the form of elongated forearm and digit bones (digits II–V) [2]. The forelimb digits II–V are dramatically elongated, where the membrane between these elongated digits is retained during development. In contrast to the forelimb digits II–V, the thumb and five digits of the hindlimb are not elongated and the interdigital tissue disappears (Fig. 1A). In addition, bats evolved unique and elongated calcars at the ankles to support the uropatagium. Membranes that encompass the elongated bones stretch to the hind limbs, and work with associated muscles to support aerial locomotion [3].

Mammalian limbs, whether bat or mouse, arise from limb buds that protrude from the embryonic body [4]. Discrepancies in limb morphogenetic mechanisms lead to homologous structures showing morphological diversification between species (Fig. 1C). In the mouse, sculpture of digit shape is accompanied by a period of programmed cell death, or apoptosis, of cells in the interdigital regions resulting in removal of interdigital soft tissue and creation of separated digits (Fig. 1B) [4]–[6]. Expression of bone morphogenic proteins (*Bmps*) is required to activate interdigital apoptosis in the mouse [7], [8]. In the developing bat wing, inhibition of *Bmp* signaling and activation of *Fgf* signaling contribute to the retention of interdigital webbing [9]. In addition to wing construction, another uniquely chiropteran structure is the calcar, a specialization of the hindlimb that supports the tailing edge of the membranes (uropatagium), which is used to help adjust the wing camber as an airfoil during flight [10], [11]. In some insectivorous bats, the calcar can also function in the capture of insects. The calcar is located near the calcaneal tuberosity, which is adjacent to, but not in contact with, the calcaneus [12]. This structure begins its development as a relatively small cartilaginous structure, composed of hyaline cartilage, and is the last cartilage condensation in skeletal development [13].

Both the wing and the calcar contribute to powered flight in bats. Many genes likely changed their expression patterns, in time and or location in comparison to other mammals, to generate these structures [14]. Our previous mRNA-Seq and WISH analyses demonstrated that all of the genes at the 5′ end of the *Hoxd* gene cluster *(Hoxd9-13*) and *Tbx3* differ in their expression patterns in the bat forelimb, where they have high and prolonged expression, compared to the hindlimbs of bats or mouse limbs [15]. Another gene that showed differential expression is the tumor suppressor gene *Fam5c*, which showed specific expression in the short digit regions. However, other genes likely also contributed to the special structures required for flight in bats by changing their expression patterns. In this study, we focused on two additional genes, *Meis2* and *Mab21l2*, which are expressed in the bat fore- and hindlimbs, and *Tbx3*, which is expressed in the calcar.

*Meis2*, also known as *Mrg1*, is a homeobox gene belonging to the TALE (Three Amino Acid Loop Extension) superclass, which consists of the *Pbx* (*Pbx1*, *Pbx2*, *Pbx3 and Pbx4*) and *Meis* (*Prep1, Prep2*, *Meis1*, *Meis2 and Meis3*) classes, and plays a key role in restricting the expression patterns of genes during embryogenesis [16], [17]. Studies of early limb development in the chicken has shown that restriction of *Meis2* expression to the proximal part of the limb is essential for limb development, and Hoxd genes contribute to restrict Meis2 expression to the proximal limb bud [18]. Expression of *Meis2* is also correlated with the proliferation of neuroblastoma cells, retinal progenitor cells (PRCs) and the formation of somatic tissue [19]–[21]. However, the expression pattern of *Meis2* in bat limbs, especially in late limb morphogenesis, is unknown. To investigate changes in the expression pattern of *Meis2* in bat limbs we compared the expression of *Meis2* in fore- and hindlimbs of both bats and mice.

*Mab21l2* is a member of the Male-abnormal 21 gene family (*Mab21* family) that is first identified as a cell fate determinant in *C. elegans* and is required for the development of sensory organs [22]–[25]. *Mab21* family members are highly conserved and show partially overlapping expression patterns during morphogenesis [23]. *Mab21l2* is expressed in the retina, mid- and hindbrain, ventral body wall, limb buds and developing digits during mouse and zebrafish development, indicating that *Mab21l2* may possess multiple functions [26]–[29]. Some functions of *Mab21l2* have been demonstrated by experiments where the expression of the *Mab21l2* gene has been abolished during specific developmental stages [30]–[32]. However, the function and expression patterns of *Mab21l2* in limb development are unclear. To investigate the pattern of expression *Mab21l2* in the bat limbs we compared the expression of this gene between fore- and hindlimbs in bats and mice.

*Tbx3* is a member of the T-box family of transcription factors, belongings to the *Tbx2/3/4/5* subfamily [33], [34]. *Tbx* genes are involved in limb initiation, by interacting with *Wnts* and *Fgfs*, and limbs position along the rostrocaudal axis, by acting through *dHand* and *Gli3*. *Tbx* genes also contribute to the specifie digit identities by modulating *Shh* and *BMP* signaling [35]–[37]. In the T-box family, *Tbx3* plays a particularly important role, as mutations in human *Tbx3* lead to limb malformations that include the total absence of the ulnar bone and digit IV [38]. Our previous studies indicated that *Tbx3* shows prolonged and high expression in the area of the bat digits that are elongated. Here in this study, we aim to validate the results from the mRNA-Seq data and to better define the exact expression pattern of *Tbx3* in the bat hindlimb.

## Materials and Methods

### Ethics Statement

The field studies did not involve endangered or protected species. All procedures involving animals were carried out in accordance with the Policy on the Care and Use of Animals, approved by the Animal Ethical Committee of East China Normal University (ID *no.* AR2012/03001).

### Sample Collection

Embryos of the common bent-wing bat (*Miniopterus schreibersii*) were collected from wild-caught, pregnant females captured in a cave, named Yulong, in Anhui province (30°20.263′ N, 117°50.180′ E) of China, from May to June 2012. Cave Yulong Tourism Development Co., Ltd. permitted bat capture in this location. All bat embryos were staged-according to the system developed by *Cretekos et al* [39]. The study by *Wang et al* [40] on prenatal development in *M. schreibersii* was also used to stage the bat embryos. Mouse embryos (ICR strain) were collected from timed matings. Noon of the day on which the vaginal plug was detected was considered to be E0.5. More precise ages of the stages was according to the Carnegie staging system [41]. Corresponding stages for mice and bats were based on our previous study [15]. Both bat and mouse embryos were fixed in 4% paraformaldehyde (PFA) in phosphate buffered saline (PBS) overnight. Fixed embryo tissues was then washed with 25, 50 and 75% methanol/PBTX solutions, and stored in 100% methanol at −80°C before being used for whole-mount in situ hybridization (WISH) [42].

### mRNA-Seq Data Reanalysis

Genome-wide mRNA sequencing had been performed on the fore- and hindlimbs of bats at stages 15–17 in our previous study [15]. The mRNA data was submitted to the Gene Expression Omnibus (GEO) database under accession number GSE50699. Reanalysis of the mRNA-Seq data was performed with the methods previously described [15] **.** The edgeR and DEGseq packages were used to normalize the data and identify genes showing differential expression among the samples [43]–[46]. Q-value less than 0.0001 were regarded as showing significant differential expression [47].

### Gene Cloning and Sequence Alignment

Embryonic S14–17 whole bat body was used for the extraction of total RNA, using the RNAiso kit (Takara, D312, Japan). A High Capacity cDNA Reverse Transcription Kit (AB applied biosystem, 4368814, USA) was used to reverse transcribe the cDNA. For *Meis2* and *Mab21l2*, pairs of primers (see Table S1) were designed to amplify the complete coding sequence. For *Tbx3*, two pairs of primers (see Table S1) were used to amplify the whole coding sequence. The Polymerase Chain Reaction (PCR) was performed with denaturation at 95°C for 5 min, followed by 35 amplification cycles, and a final extension at 72°C for 10 min. PCR products were isolated through 1% agarose gels and purified with Agarosel Gel DNA Extraction Kits (Takara, AK801, Japan), ligated into pGEM-T easy vector (Promega, 28521916, USA), cloned and sequenced using the Terminator kits (Applied Biosystems) on an ABI 3730 DNA sequencer. The obtained sequences of *Meis2, Mab21l2* and *Tbx3* of bat were aligned with the open reading frames (ORF) of the mouse *Meis2* (NM_001136072.2), *Mab21l2* (NM_011839.3) and *Tbx3* (NM_198052.2) sequences, respectively, using the ClustalW method implemented in MEGA5 [48]. Protein sequences encoded by *Meis2, Mab21l2* and *Tbx3* from the bat (*M. schreibersii*) were separately aligned with 20, 21 and 14, respectively, sequences from diverse mammals (Fig. S1–S3 and Table S2). Newly obtained CDS sequences of *Meis2, Mab21l2* and *Tbx3* of *M. schreibersii* were deposited into GenBank with accession numbers KJ670370, KJ670371 and KJ6703702.

### Gene Evolutionary Analysis

Tajima relative rate tests [49] implemented in MEGA5 [48], were performed using amino acid sequences to investigating the relative rates of evolutionary change in *Tbx3* of bats and 12 other mammalian species, with the human, pig or mouse sequence used as outgroups. A χ2 test statistic of greater than 3.841 indicates statistical significance (P<0.05), and accelerated evolution, and was used to reject the null hypothesis of equal rates between tested lineages [49].

Tests for positive selection in *Tbx3* were conducted using the PAML package [50]. We conducted the branch models (free-ratio, one-ratio, two-ratio and three-ratio model tests) for *Tbx3* to test if positive selection acted on the common ancestral branch of bats. The tree topology used for these molecular evolutionary analyses was based on the currently accepted phylogenetic relationships [51]–[54]. CODEML, from the PAML package, was used to estimate the rates of synonymous (d_S_) and nonsynonymous (d_N_) substitutions, and the d_N_/d_S_ ratio (omega, ω) [50]. In the two-ratio models, the common ancestors of the bats and rodents were separately set as the foreground, with the other mammalian branches set as background. In the three-ratio model, the bat common ancestor and the rodent common ancestor were set as foreground, with the other mammalian branches being background. Results for the alternative and null hypotheses were compared using likelihood ratio tests (LRTs). To test the selection pressures at codon sites of the bat and mouse *Tbx3*, the site model and branch site model were also performed using PAML software.

### Whole-mount in situ hybridization (WISH)

WISH was performed by the method previously described [42]. Digoxygenin-labeled RNA derived from bat and mouse sequences were used as probes. For the bat, we designed and obtained a 491 bp *Meis2* fragment, a 550 bp *Mab21l2* fragment and a 543 bp *Tbx3* fragment by RT-PCR from total forelimb RNA from a bat embryo, which were cloned into PGEM-T vectors. For the mouse, *Meis2*, *Mab21l2* and *Tbx3* (604 bp, 744 bp and 609 bp, respectively) fragments were obtained from total RNA from mouse embryos by RT-PCR and cloned into PGEM-T vectors. Primers used for amplifying these fragments are listed in Table S1. Plasmids were linearized with the restriction enzymes NcoI or SpeI (Fermentas, 00104053 and 00108276, USA). Linearized vectors were transcribed with T7 or SP6 polymerase (Fermentas, 00120690 and 00106529, USA) to synthesize the digoxygenin-labeled RNA probes (Roche, 11277073910, Switzerland). RNA probes were used at a concentration of about 2 µg/ml. Embryos were incubated at 68°C to hybridize overnight, stained with nitro blue tetrazolium/5-bromo-4-chloro-3-indolylphosphate (NBT/BCIP) in the dark (Roche, 11697471001, Switzerland). When NBT/BCIP staining developed to the desired extent, embryonic samples were washed in NTMT and then PBS, postfixed in 4% PFA and washed in a gradient of methanol/PBTX solutions to 100% methanol. Embryos were photographed by a Leica stereo-micro scope S8 APO.

## Results

### Significant Differential Expression of *Meis2* and *Mab21l2* in Bat Fore- and Hindlimbs

**Figure 2:**
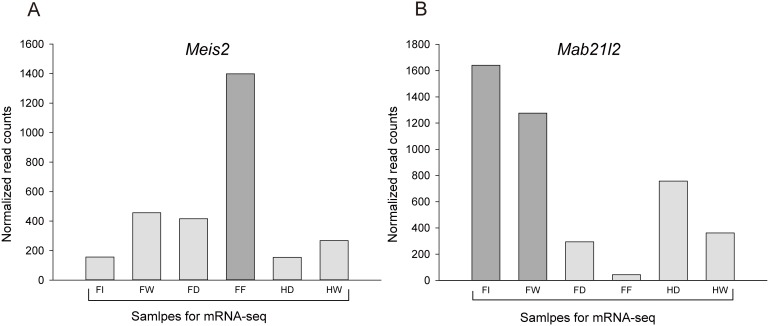
Comparison of the expression levels of *Meis2, Mab21l2* in mRNA-Seq samples at bat embryonic stages 15–17. FI: forelimb digit I; FW: interdigital tissues between forelimb digits I and II; FD: elongating forelimb digits II–V; FF: interdigital tissues between forelimb digits II and V; HD: hindlimb digits I–V; HW: interdigital tissues between hindlimb digits I and V.

At stage 15, the bat forelimb begins to condense and elongate, a process that continues through later stages [40]. Our previous study applied mRNA-Seq to find genes that may participate in digit elongation and/or interdigital tissue retention in the fore- and hindlimbs of bats at stages 15 to 17 [15]. This study compared mRNA-Seq data from elongating forelimb digits to that from short digits (thumb and hindlimb digits) and identified seven genes that displayed significant differential expression patterns, which were then examined in more detail. A reanalysis of this mRNA-Seq data identified two additional genes (*Meis2* and *Mab21l2*) that are also expressed at significantly different levels (q<0.0001) in these tissues (Fig. 2).

Comparing all digits and interdigital tissues, *Meis2* is most highly expressed in the interdigital tissues of the elongating forelimb digits (digits II–V), where tissues are retained and form the wing membrane in the adult bat (Fig. 2A). Interdigital tissues of the digits that remain short, where separated digits (thumb and hindlimb digits) form, show only slight levels of expression. All of the digits, whether elongating forelimb digits or the remaining short digits, show low levels of *Meis2* expression. *Mab21l2* shows a different pattern and is most highly expressed in forelimb digit I and the interdigital tissues between forelimb digits I–II, where the thumb will form and the interdigital tissues will recede (Fig. 2B). Forelimb digits II–V, and their interdigital tissues, show lower levels of *Mab21l2* expression, where the interdigital tissues, especially, show the lowest expression. Expression of *Mab21l2* in the hindlimb digits, and their interdigital tissues, is detectable, but at levels lower than those of the short forelimb digit (thumb) and its interdigital tissues.

### Prolonged and High Expression of *Meis2* in the Elongating Wing Area of Bats

To validate the mRNA-Seq results, we conducted WISH in a continuous series of embryonic stages, including those before and after the hindlimb interdigital tissue regression that allows the formation of the five free digits on each foot [39]. Our results are consistent with the mRNA-Seq data. We also examined embryonic stages in the mouse that correspond to those of the bat, where most of the interdigital tissues disappear and the free digits form [55], [56].

**Figure 3:**
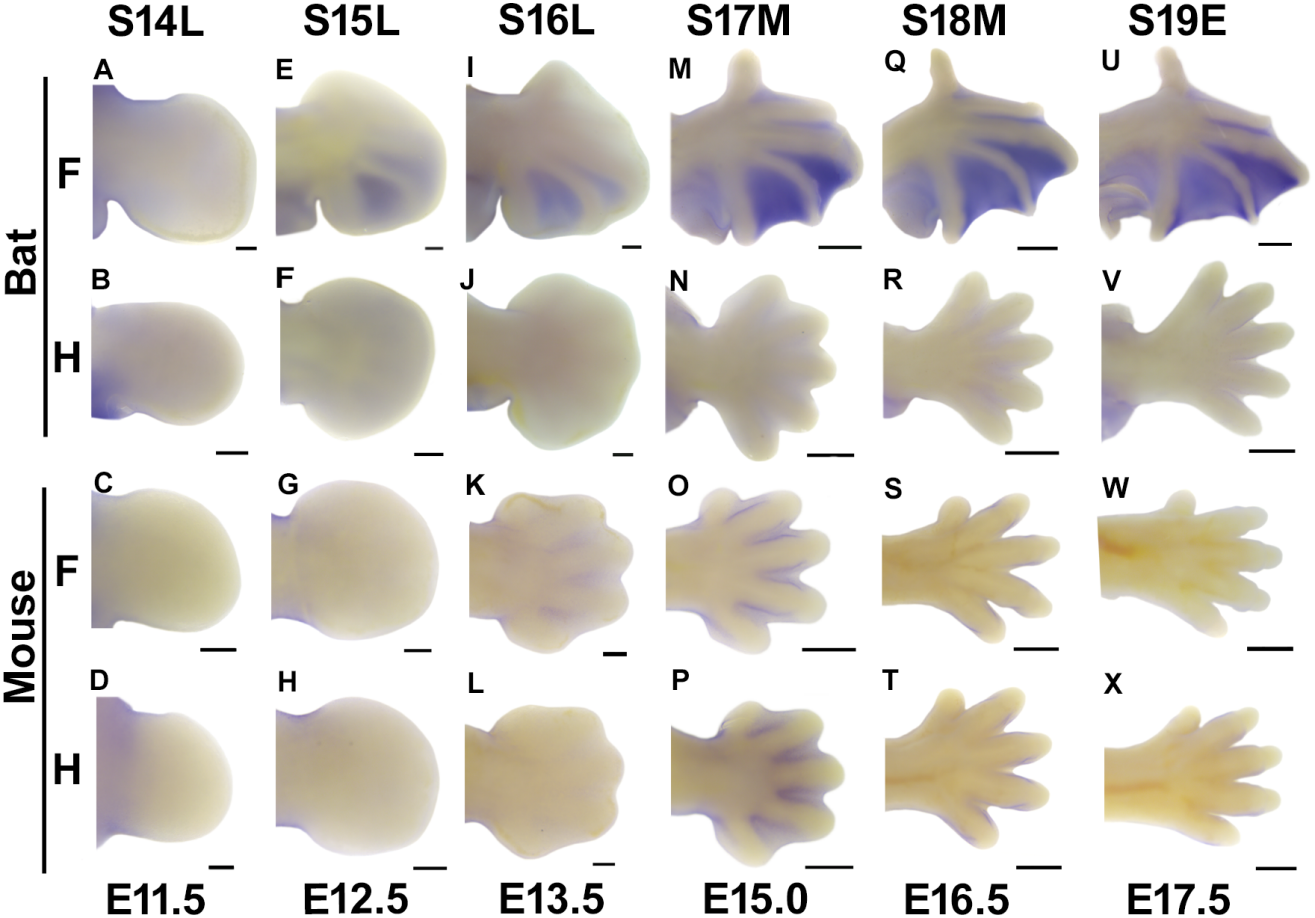
*Meis2* expression in the fore- (F) and hindlimbs (H) of embryonic bats and mice visualized by WISH. All images are the dorsal view with anterior pointing up.S14L: Late stage14; S17M: Middle stage 17; S19E: Early stage 19; Scale bars, 200 µm in (A–L) and 500 µm in (M–X).

In the bat wing, *Meis2* shows a unique and high expression pattern. At stage 14, *Meis2* is not expressed in the forelimbs (Fig. 3A). However, from stage 15 to stage 16, *Meis2* starts being expressed in the interdigital tissues between digits III–V (Fig. 3E and I). At stage 17, *Meis2* extends its expression area to the anterior of the forelimb and is expressed at a high level in interdigits II–V and a low level in the interdigits I–II, where interdigital tissues are receding (Fig. 3M). The area and level of *Meis2* expression in the forelimbs persists until stage 19 (Fig. 3Q and U).

In the bat hindlimbs, *Meis2* shows very little expression through the investigated stages (Fig. 3). In the mouse fore- and hindlimbs, the expression patterns are similar to those of the bat hindlimb, with exceptions at E15. At embryonic day 15, *Meis2* is expressed in all of the interdigital tissues of the fore- and hindlimbs (Fig. 3O and P).

### Diversified Expression of *Mab21l2* in Bat and Mouse Fore- and Hindlimbs

**Figure 4:**
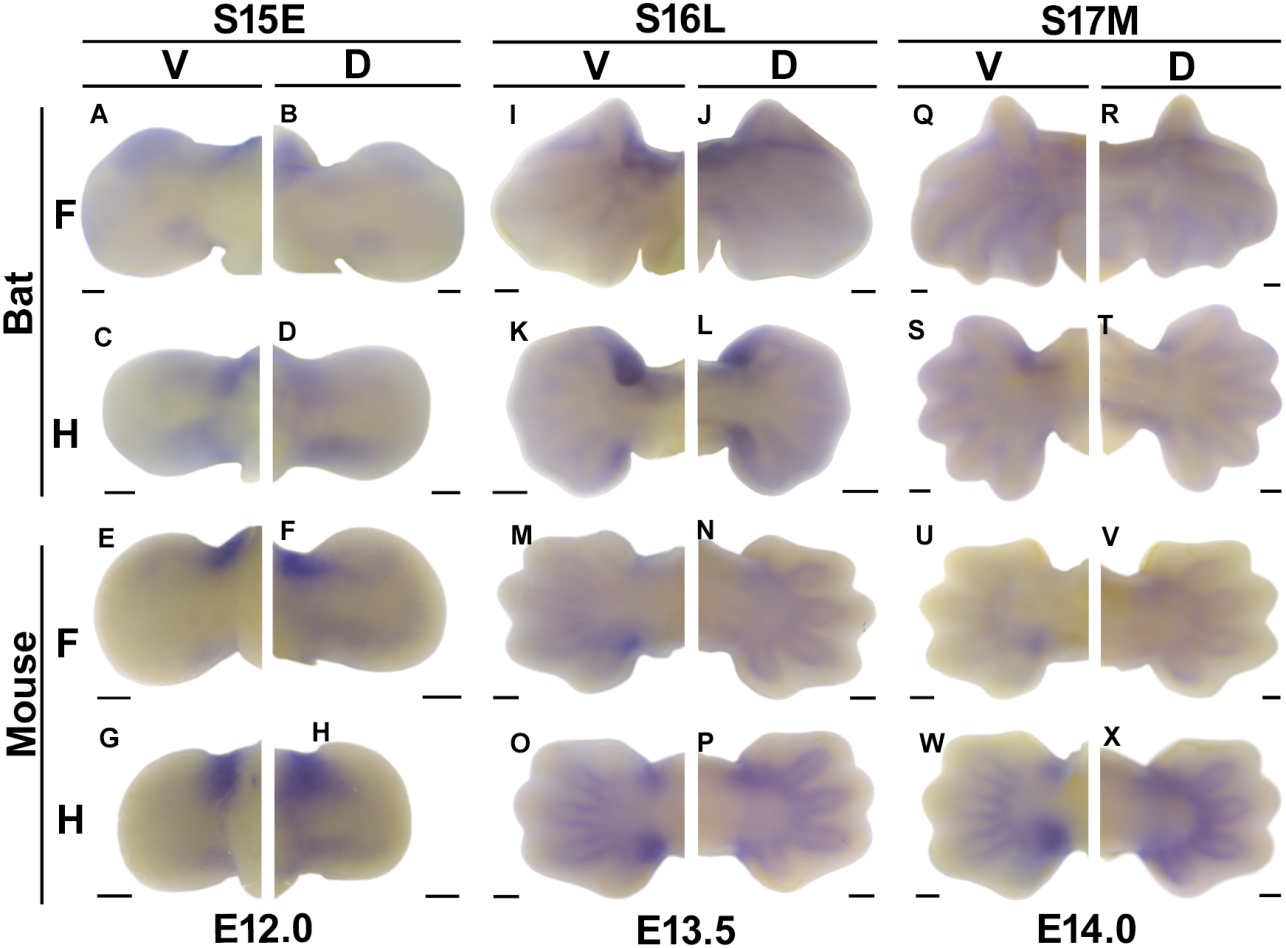
*Mab21l2* expression in the fore- (F) and hindlimbs (H) of embryonic bats and mice visualized by WISH. Each stage is shown with dorsal (D) and ventral (V) views. Anterior is up in all images. Scale bar, 200 µm.

Our WISH results for *Mab21l2* are generally consistent with the mRNA-Seq data from the bat. At bat stage 15, when digits are condensing in the forelimb, expression of *Mab21l2* is very low at the two ends of the hand and foot plates, where the wrists and ankles will form (Fig. 4A–D). In the mouse, *Mab21l2* is highly expressed at the anterior ends of the hand and foot plates, where the wrists and ankles will form, with low expression at the posterior ends of the hand- and foot plates (Fig. 4E–H). Additionally, expression of *Mab21l2* does not display a distinct dorsal-ventral difference in the bat or the mouse (Fig. 4A–H).

Expression of *Mab21l2* displays some differences between the dorsal and ventral views in both the bat at stage 16 and the mouse at the corresponding stage (Fig. 4I–P). The expression positions are similar in these two views and the ventral view shows a higher expression level and a larger expression domain. In the bat, *Mab21l2* is highly expressed in the interdigital tissues of digits I–II and in the wrist on the anterior side, and not expressed in the posterior side (Fig. 4I–J). In the hindlimb, expression of *Mab21l2* is at the two ends of the ankle, with a higher expression level on the anterior side compared to the posterior (Fig. 4K–L). In the mouse, *Mab21l2* is expressed in the perichondrium of digits II–V of both the fore- and hindlimb at E13.5, when the phalanges begin to form and the interdigital tissues disappear (Fig. 4M–P). *Mab21l2* is expressed at both ends of the wrist and the ankle, with higher expression in the posterior end (Fig. 4M–P).

At bat stage 17 expression of *Mab21l2* becomes weak in the fore- and hindlimbs (Fig. 4Q–T). In the mouse, expression of *Mab21l2* at E14 is consistent with that seen at E13.5, but becomes weak in the forelimb (Fig. 4U–X) and remains strong in the hindlimbs.

There are two main differences in the expression patterns of *Mab21l2* between bats and mice. First, *Mab21l2* is only expressed at the anterior end of the bat wrist, but is expressed at both ends of the mouse wrist, as well as at both ends of the ankle in bat and mouse hindlimbs. The second difference is that *Mab21l2* is almost not expressed in the perichondrium of the digits in the bat, but has distinct expression in the mouse.

### *Tbx3* Expression in the Bat Calcar

**Figure 5:**
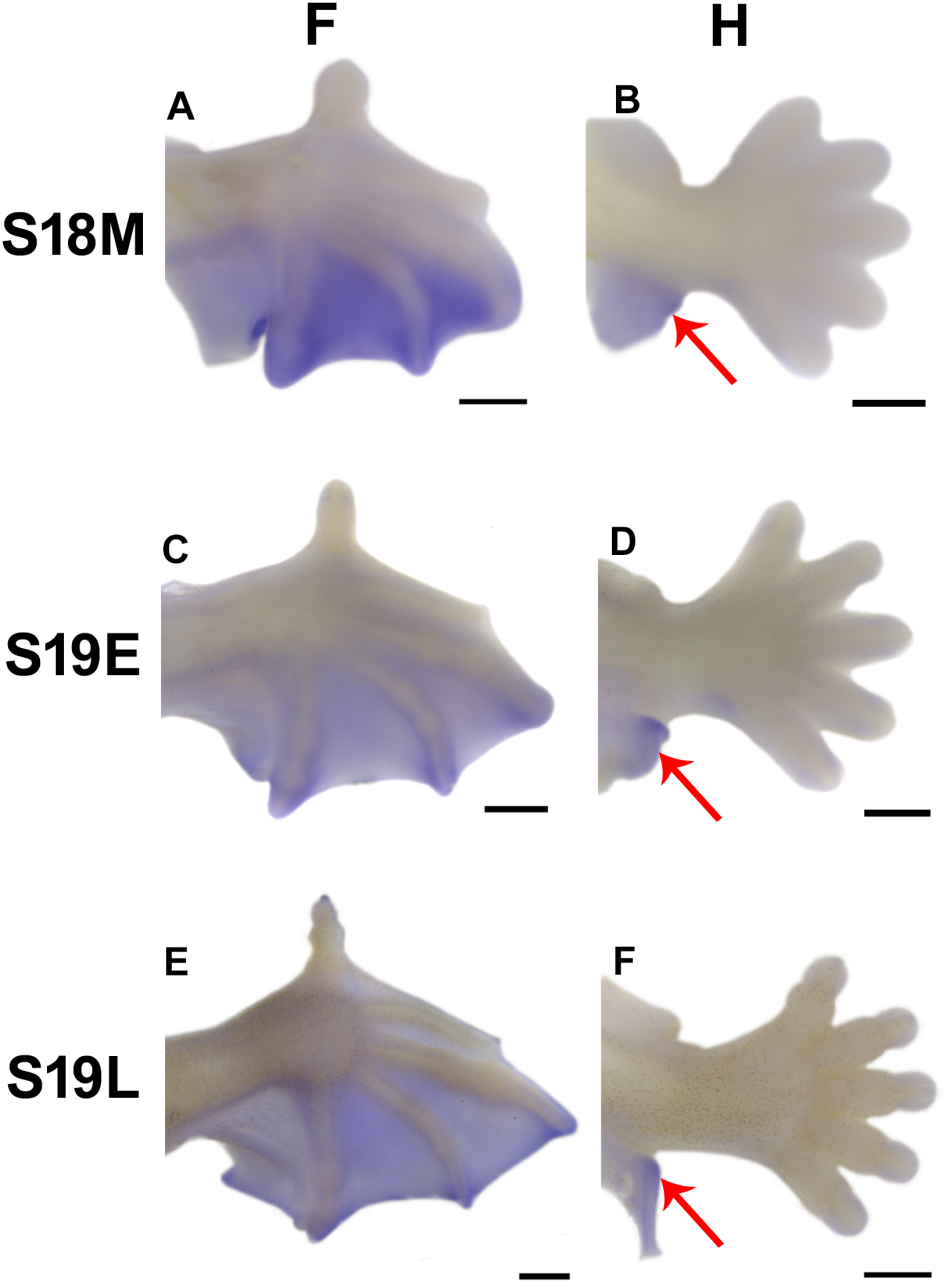
*Tbx3* expression in the fore- (F) and hindlimbs (H) of embryonic bats visualized by WISH. All images are the dorsal view with anterior pointing up. Scale bar, 500 µm.

WISH was used to validate the mRNA-Seq expression results for *Tbx3* for bat stages 18 to 19 when the calcar appears and becomes distinct [39]. At stage 18, expression of *Tbx3* is consistent with our previous in situ hybridization results, with high expression in the elongating interdigital areas (Fig. 5A). The expression pattern of *Tbx3* in the forelimb is sustained through the investigated stages, although with some reduction in expression levels (Fig. 5C and E). In the hindlimb, distinct *Tbx3* expression is found in the area of the calcar, a unique structure at the ankle, where in stages 18–19 *Tbx3* expression is high and prominent, as indicated by the red arrow in Figure 5 (B, D and F). The mouse, which does not have a structure homologous to the calcar in the ankle, does not show *Tbx3* expression in the comparable area when the mouse is examined at comparable stages (Figures not shown).

### Alignment and Evolutionary Analyses of *Meis2, Mab21l2* and *Tbx3*

We amplified, cloned and sequenced the complete coding sequences (CDS) of the *Meis2, Mab21l2* and *Tbx3* from the bat *M. schreibersii*. To examine changes in the sequences of *Meis2, Mab21l2* and *Tbx3* in bats, the amino acid sequences encoded by these genes were aligned with those from the mouse and other mammalian species (Figs. S1–S3). The bat *Meis2, Mab21l2* and *Tbx3* protein sequences were 99.7%, 100% and 93.8% identical to those from the mouse, respectively. The amino acid sequence alignments suggest that *Meis2* and *Mab21l2* share high levels of sequence identity among mammals. Bat *Tbx3* shares high sequence identity with the *Tbx3* sequences of other mammals in its DNA-binding domain, but lower levels in its transcription repression domain (Fig. S3). To examine the rates of evolution of *Tbx3* in bats, we applied the Tajima relative rate test to the protein sequences from pairs of mammals, using orthologous sequences from human, pig or mouse as outgroups (Table S3). Results of the relative rate tests indicate *Tbx3* evolves at a significantly different rate in bats compared with most other mammals (χ^2^>3.841, P<0.05). To examine whether the *Tbx3* was driven by a high evolutionary rate, a molecular evolutionary analysis for positive selection on *Tbx3* from diverse mammals was conducted (Table S4). Likelihood ratio tests (LRTs) for all models (including the branch models, site model and branch site models) failed to find evidence for positive selection, suggesting that negative selection predominated and consistent with the functions of the gene being conserved in bat and mouse (Table S4).

## Discussion

*Meis2* and *Mab21l2* have highly conserved amino acid sequences, suggesting that the proteins encoded by *Meis2* and *Mab21l2* have not changed much in mammals, as their functions are very important. However, these two genes show distinct expression patterns in bats and mice during limb development. Although *Tbx3* shows a higher evolutionary rate in bats in relative rate tests, LRTs did not find evidence for positive selection, which suggests that the results are consistent with gene function being conserved. We infer that the high expression of *Tbx3* in the bat calcar contributes to the development of the calcar. These observations support the hypothesis that the divergent morphology of mammalian limbs was acquired through modulation of the spatiotemporal expression patterns of many functionally conserved genes.

The prolonged and high expression of *Meis2* in the interdigital tissues between the elongated bat digits suggests that it participates in the sculpting of the bat wings. *Meis2* is known to play diverse roles in morphogenesis, including roles in the lens, pancreas and cardiac septum [57]–[59]. Studies on members of the *Meis* family have shown that *Meis3* can suppress cellular apoptosis and that *Meis1* and *Meis2* promote cellular proliferation and differentiation [20], [21], [60]. Here we propose that *Meis2* participates in the retention of interdigital tissues between the elongated digits in the bat, thereby forming the webbing of the bat wing. Though *Meis2* shows some expression in the interdigital tissues of the mouse, this expression is much lower than that seen in the bat wing, suggesting a much stronger biological function in the bat wing than in the mouse limbs. Overexpression of *Meis1* in limbs, which leads to low *msx1* expression, results in the persistence of interdigital membranes [61]. Low expression of *Meis2* in the mouse limbs may not be sufficient to allow the persistence of interdigital tissues in the fore- and hindlimbs, while the higher expression in the bat wing is sufficient to retain the interdigital tissues between the elongated digits.

*Mab21l2* acts as a repressor that can complement the effect of *Bmp4* on the formation of the dorsal-ventral (D–V) axis in *Xenopus* [62], [63]. In the limbs of the bat and the mouse, expression of *Mab21l2* shows differential expression on the dorsal and ventral surfaces, suggesting that this gene may play some role in D–V axis patterning in the limb. Dramatic changes occur to the anterior-posterior (A–P) axis of the limb plate when the bat forms its asymmetric forelimb. In the bat, *Mab21l2* shows restricted high expression at the anterior area of the hand and differential A–P expression in the fore- and hindlimbs, suggesting that this gene may participate in the asymmetric A–P axis patterning of the bat wing. *Mab21l2* improves cell proliferation, differentiation and prevents apoptosis [30], [32] and it is expressed at the wrists and ankles, suggesting that *Mab21l2* may contribute to the formation of the wrist and the ankle. *Mab21l2* is highly expressed in the perichondrium of mouse digits and this expression pattern is not observed in the bat, suggesting that this gene may have a different role in mouse digit development. Additional studies are required to clarify the functions of *Mab21l2* in limb formation.

During bone morphogenesis, *Tbx3* plays a pivotal role in osteogenic differentiation and promotes proliferation and suppresses osteoblasts differentiation by increasing its expression [64]. *Tbx3* is expressed in the bat hindlimb where the calcar is forming, suggests that it is involved in the formation of calcar. We propose that *Tbx3* may act on bat calcar formation by regulating chondrocyte proliferation and osteogenic differentiation. In addition, changes in the *Tbx3* protein sequence detected in the transcriptional repression domain may result in differences in transcriptional efficiency among mammals [65].

In conclusion, our results demonstrate differential expression patterns for three genes with high sequences identity, consistent with genes functions being conserved, during bat and mouse limb development. *Meis2* may function to sculpt bat wing membranes, *Mab21l2* may participate in the D-V and A-P patterning of limbs, and *Tbx3* may contribute to the formation of the calcar. Limbs, as homologous structures, in mammals show diversity in morphology. Differences in the expression patterns of functionally conserved genes, leading to changes in morphogenetic mechanisms, during limb development may be the most significant mechanism in the evolution of differing limb morphologies in mammals.

## Supporting Information

Figure S1 **Alignment of amino acid sequences of** ***Meis2*** **in mammals.**
(PDF)

Figure S2 **Alignment of amino acid sequences of** ***Mab21l2*** **in mammals.**
(PDF)

Figure S3 **Alignment of amino acid sequences of** ***Tbx3*** **and species topologies of mammals.** (A) Alignment of amino acid sequences of *Tbx3* in mammals. The protein domains of *Tbx3* were referred to the prediction of mouse *Tbx3* from Universal Protein Resource (http://www.uniprot.org/uniprot/P70324). (B) Species topologies of mammals used in the molecular evolutionary analysis of *Tbx3*.
(PDF)

Table S1 **Primers for making WISH probes and for amplifying complete CDS of Bat** ***Meis2*** **,** ***Mab21l2*** **and** ***Tbx3*** **.**
(PDF)

Table S2 **Information on species examined in amino acid alignment and molecular evolutionary analysis.**
(PDF)

Table S3 **Tajima relative rate tests of** ***Tbx3*** **in mammls.**
(PDF)

Table S4 **Branch model tests of selection pressure on** ***Tbx3*** **gene in bats and rodent.**
(PDF)
