# Supplementary material for: Differential Expression of Meis2, Mab21l2 and Tbx3 during Limb Development Associated with Diversification of Limb Morphology in Mammals
Source: PLoS One. 2014 Aug 28;9(8):e106100. doi: 10.1371/journal.pone.0106100 (PMC4148388; doi:10.1371/journal.pone.0106100)
Supplement: Figure S1 — Alignment of amino acid sequences of Meis2 in mammals. (PDF) [file pone.0106100.s001.pdf]

Figure S1. Alignment of amino acid sequences of *Meis2* in mammals.

```
                                                                                                              1 1111111111 1111111111 1111111111 1111111111 1111111111 1111111111 1111111111 1111111111 1111111111 1111111
                              1 1111111112 2222222223 3333333334 4444444445 5555555556 6666666667 7777777778 8888888889 9999999990 0000000001 1111111112 2222222223 3333333334 4444444445 5555555556 6666666667 7777777778 8888888889 9999999
                     1234567890 1234567890 1234567890 1234567890 1234567890 1234567890 1234567890 1234567890 1234567890 1234567890 1234567890 1234567890 1234567890 1234567890 1234567890 1234567890 1234567890 1234567890 1234567890 1234567
M.musculus           MAQRYDELPH YGGMDGVGVP ASMYGDPHAP RPIPPVHHLN HGPPLHATQH YGAHAPHPNV MPASMGSAVN DALKRDKDAI YGHPLFPLLA LVFEKCELAT CTPREPGVAG GDVCSSDSFN EDIAVFAKQV RAEKPLFSSN PELDNLMIQA IQVLRFHLLE LEKVHELCDN FCHRYISCLK GKMPIDLVID ERDGSSK
M. schreibersii      .......... .......... .......... .......... .......... .......... .......... .......... .......... .......... .......... .......... .......... .......... .......... .......... .......... .......... .......... .......
M.lucifugus          .......... .......... .......... .......... .......... .......... .......... .......... .......... .......... .......... .......... .......... .......... .......... .......... .......... .......... .......... .......
E.caballus           .......... .......... .......... .......... .......... .......... .......... .......... .......... .......... .......... .......... .......... .......... .......... .......... .......... .......... .......... .......
B.mutus              .......... .......... .......... .......... .......... .......... .......... .......... .......... .......... .......... .......... .......... .......... .......... .......... .......... .......... .......... .......
C.simum              .......... .......... .......... .......... .......... .......... .......... .......... .......... .......... .......... .......... .......... .......... .......... .......... .......... .......... .......... .......
O.orca               .......... .......... .......... .......... .......... .......... .......... .......... .......... .......... .......... .......... .......... .......... .......... .......... .......... .......... .......... .......
P.hodgsonii          .......... .......... .......... .......... .......... .......... .......... .......... .......... .......... .......... .......... .......... .......... .......... .......... .......... .......... .......... .......
C.lupus familiaris   .......... .......... .......... .......... .......... .......... .......... .......... .......... .......... .......... .......... .......... .......... .......... .......... .......... .......... .......... .......
O.rosmarus divergens .......... .......... .......... .......... .......... .......... .......... .......... .......... .......... .......... .......... .......... .......... .......... .......... .......... .......... .......... .......
H.sapiens            .......... .......... .......... .......... .......... .......... .......... .......... .......... .......... .......... .......... .......... .......... .......... .......... .......... .......... .......... .......
P.anubis             .......... .......... .......... .......... .......... .......... .......... .......... .......... .......... .......... .......... .......... .......... .......... .......... .......... .......... .......... .......
P.troglodytes        .......... .......... .......... .......... .......... .......... .......... .......... .......... .......... .......... .......... .......... .......... .......... .......... .......... .......... .......... .......
N.leucogenys         .......... .......... .......... .......... .......... .......... .......... .......... .......... .......... .......... .......... .......... .......... .......... .......... .......... .......... .......... .......
C.jacchus            .......... .......... .......... .......... .......... .......... .......... .......... .......... .......... .......... .......... .......... .......... .......... .......... .......... .......... .......... .......
H.glaber             .......... .......... .......... .......... .......... .......... .......... .......... .......... .......... .......... .......... .......... .......... .......... .......... .......... .......... .......... .......
O.princeps           .......... .......... .......... .......... .......... .......... .......... .......... .......... .......... .......... .......... .......... .......... .......... .......... .......... .......... .......... .......
M.ochrogaster        .......... .......... .......... .......... .......... .......... .......... .......... .......... .......... .......... .......... .......... .......... .......... .......... .......... .......... .......... .......
I.tridecemlineatus   .......... .......... .......... .......... .......... .......... .......... .......... .......... .......... .......... .......... .......... .......... .......... .......... .......... .......... .......... .......
C.porcellus          .......... .......... .......... .......... .......... .......... .......... .......... .......... .......... .......... .......... .......... .......... .......... .......... .......... .......... .......... .......
```

```
                     1122222222 2222222222 2222222222 2222222222 2222222222 2222222222 2222222222 2222222222 2222222222 2222222222 2233333333 3333333333 3333333333 3333333333 3333333333 3333333333 3333333333 3333333333 3333333333 3333333
                     9900000000 0011111111 1122222222 2233333333 3344444444 4455555555 5566666666 6677777777 7788888888 8899999999 9900000000 0011111111 1122222222 2233333333 3344444444 4455555555 5566666666 6677777777 7788888888 8899999
                     8901234567 8901234567 8901234567 8901234567 8901234567 8901234567 8901234567 8901234567 8901234567 8901234567 8901234567 8901234567 8901234567 8901234567 8901234567 8901234567 8901234567 8901234567 8901234567 8901234
M.musculus           SDHEELSGSS TNLADHNPSS WRDHDDATST HSAGTPGPSS GGHASQSGDN SSEQGDGLDN SVASPGTGDD DDPDKDKKRQ KKRGIFPKVA TNIMRAWLFQ HLTHPYPSEE QKKQLAQDTG LTILQVNNWF INARRRIVQP MIDQSNRAVS QGAAYSPEGQ PMGSFVLDGQ QHMGIRPAGP MSGMGMNMGM DGQWHYM
M. schreibersii      .......... .......... .......... .......... .......... .......... .......... .......... .......... .......... .......... .......... .......... .......... .......... .......... .......... .......... .......... .......
M.lucifugus          .......... ........A. .......... .......... .......... .......... .......... .......... .......... .......... .......... .......... .......... .......... .......... .......... .......... .......... .......... .......
E.caballus           .......... ........A. .......... .......... .......... .......... .......... .......... .......... .......... .......... .......... .......... .......... .......... .......... .......... .......... .......... .......
B.mutus              .......... ........A. .......... .......... .......... .......... .......... .......... .......... .......... .......... .......... .......... .......... .......... .......... .......... .......... .......... .......
C.simum              .......... ........A. .......... .......... .......... .......... .......... .......... .......... .......... .......... .......... .......... .......... .......... .......... .......... .......... .......... .......
O.orca               .......... ........A. .......... .......... .......... .......... .......... .......... .......... .......... .......... .......... .......... .......... .......... .......... .......... .......... .......... .......
P.hodgsonii          .......... ........A. .......... .......... .......... .......... .......... .......... .......... .......... .......... .......... .......... .......... .......... .......... .......... .......... .......... .......
C.lupus familiaris   .......... ........A. .......... .......... .......... .......... .......... .......... .......... .......... .......... .......... .......... .......... .......... .......... .......... .......... .......... .......
O.rosmarus divergens .......... ........A. .......... .......... .......... .......... .......... .......... .......... .......... .......... .......... .......... .......... .......... .......... .......... .......... .......... .......
H.sapiens            .......... .......... .......... .......... .......... .......... .......... .......... .......... .......... .......... .......... .......... .......... .......... .......... .......... .......... .......... .......
P.anubis             .......... .......... .......... .......... .......... .......... .......... .......... .......... .......... .......... .......... .......... .......... .......... .......... .......... .......... .......... .......
P.troglodytes        .......... .......... .......... .......... .......... .......... .......... .......... .......... .......... .......... .......... .......... .......... .......... .......... .......... .......... .......... .......
N.leucogenys         .......... .......... .......... .......... .......... .......... .......... .......... .......... .......... .......... .......... .......... .......... .......... .......... .......... .......... .......... .......
C.jacchus            .......... .......... .......... .......... .......... .......... .......... .......... .......... .......... .......... .......... .......... .......... .......... .......... .......... .......... .......... .......
H.glaber             .......... .......... .......... .......... .......... .......... .......... .......... .......... .......... .......... .......... .......... .......... .......... .......... .......... .......... .......... .......
O.princeps           .......... .......... .......... .......... .......... .......... .......... .......... .......... .......... .......... .......... .......... .......... .......... .......... .......... .......... .......... .......
M.ochrogaster        .......... ........A. .......... .......... .......... .......... .......... .......... .......... .......... .......... .......... .......... .......... .......... .......... .......... .......... .......... .......
I.tridecemlineatus   .......... .......... .......... .......... .......... .......... .......... .......... .......... .......... .......... .......... .......... .......... .......... .......... .......... .......... .......... .......
C.porcellus          .......... .......... .......... .......... .......... .......... .......... .......... .......... .......... .......... .......... .......... .......... .......... .......... .......... .......... .......... .......
```
