# Supplementary material for: Differential Expression of Meis2, Mab21l2 and Tbx3 during Limb Development Associated with Diversification of Limb Morphology in Mammals
Source: PLoS One. 2014 Aug 28;9(8):e106100. doi: 10.1371/journal.pone.0106100 (PMC4148388; doi:10.1371/journal.pone.0106100)
Supplement: Figure S2 — Alignment of amino acid sequences of Mab21l2 in mammals. (PDF) [file pone.0106100.s002.pdf]

Figure S2. Alignment of amino acid sequences of *Mab21l2* in mammals.

```
                                                                                                                           1 1111111111 1111111111 1111111111 1111111111 1111111111 1111111111 1111111111 1111111111 1111111111 1111111
                            1 1111111112 2222222223 3333333334 4444444445 5555555556 6666666667 7777777778 8888888889 9999999990 0000000001 1111111112 2222222223 3333333334 4444444445 5555555556 6666666667 7777777778 8888888889 9999999
                   1234567890 1234567890 1234567890 1234567890 1234567890 1234567890 1234567890 1234567890 1234567890 1234567890 1234567890 1234567890 1234567890 1234567890 1234567890 1234567890 1234567890 1234567890 1234567890 1234567
M.musculus         MIAAQAKLVY QLNKYYTERC QARKAAIAKT IREVCKVVSD VLKEVEVQEP RFISSLSEID ARYEGLEVIS PTEFEVVLYL NQMGVFNFVD DGSLPGCAVL KLSDGRKRSM SLWVEFITAS GYLSARKIRS RFQTLVAQAV DKCSYRDVVK MIADTSEVKL RIRERYVVQI TPAFKCTGIW PRSAAQWPMP HIPWPGP
M. schreibersii    .......... .......... .......... .......... .......... .......... .......... .......... .......... .......... .......... .......... .......... .......... .......... .......... .......... .......... .......... .......
M.lucifugus        .......... .......... .......... .......... .......... .......... .......... .......... .......... .......... .......... .......... .......... .......... .......... .......... .......... .......... .......... .......
O.orca             .......... .......... .......... .......... .......... .......... .......... .......... .......... .......... .......... .......... .......... .......... .......... .......... .......... .......... .......... .......
S.scrofa           .......... .......... .......... .......... .......... .......... .......... .......... .......... .......... .......... .......... .......... .......... .......... .......... .......... .......... .......... .......
E.caballus         .......... .......... .......... .......... .......... .......... .......... .......... .......... .......... .......... .......... .......... .......... .......... .......... .......... .......... .......... .......
C.hircus           .......... .......... .......... .......... .......... .......... .......... .......... .......... .......... .......... .......... .......... .......... .......... .......... .......... .......... .......... .......
B.taurus           .......... .......... .......... .......... .......... .......... .......... .......... .......... .......... .......... .......... .......... .......... .......... .......... .......... .......... .......... .......
C.lupus familiaris .......... .......... .......... .......... .......... .......... .......... .......... .......... .......... .......... .......... .......... .......... .......... .......... .......... .......... .......... .......
H.sapiens          .......... .......... .......... .......... .......... .......... .......... .......... .......... .......... .......... .......... .......... .......... .......... .......... .......... .......... .......... .......
P.troglodytes      .......... .......... .......... .......... .......... .......... .......... .......... .......... .......... .......... .......... .......... .......... .......... .......... .......... .......... .......... .......
M.mulatta          .......... .......... .......... .......... .......... .......... .......... .......... .......... .......... .......... .......... .......... .......... .......... .......... .......... .......... .......... .......
P.anubis           .......... .......... .......... .......... .......... .......... .......... .......... .......... .......... .......... .......... .......... .......... .......... .......... .......... .......... .......... .......
S.boliviensis      .......... .......... .......... .......... .......... .......... .......... .......... .......... .......... .......... .......... .......... .......... .......... .......... .......... .......... .......... .......
O.garnettii        .......... .......... .......... .......... .......... .......... .......... .......... .......... .......... .......... .......... .......... .......... .......... .......... .......... .......... .......... .......
R.norvegicus       .......... .......... .......... .......... .......... .......... .......... .......... .......... .......... .......... .......... .......... .......... .......... .......... .......... .......... .......... .......
M.ochrogaster      .......... .......... .......... .......... .......... .......... .......... .......... .......... .......... .......... .......... .......... .......... .......... .......... .......... .......... .......... .......
H.glaber           .......... .......... .......... .......... .......... .......... .......... .......... .......... .......... .......... .......... .......... .......... .......... .......... .......... .......... .......... .......
C.porcellus        .......... .......... .......... .......... .......... .......... .......... .......... .......... .......... .......... .......... .......... .......... .......... .......... .......... .......... .......... .......
S.harrisii         .......... .......... .......... .......... .......... .......... .......... .......... .......... .......... .......... .......... .......... .......... .......... .......... .......... .......... .......... .......
O.princeps         .......... .......... .......... .......... .......... .......... .......... .......... .......... .......... .......... .......... .......... .......... .......... .......... .......... .......... .......... .......

                   1122222222 2222222222 2222222222 2222222222 2222222222 2222222222 2222222222 2222222222 2222222222 2222222222 2233333333 3333333333 3333333333 3333333333 3333333333 3333333333 33
                   9900000000 0011111111 1122222222 2233333333 3344444444 4455555555 5566666666 6677777777 7788888888 8899999999 9900000000 0011111111 1122222222 2233333333 3344444444 4455555555 55
                   8901234567 8901234567 8901234567 8901234567 8901234567 8901234567 8901234567 8901234567 8901234567 8901234567 8901234567 8901234567 8901234567 8901234567 8901234567 8901234567 89
M.musculus         NRVAEVKAEG FNLLSKECYS LTGKQSSAES DAWVLQFGEA ENRLLMGGCR NKCLSVLKTL RDRHLELPGQ PLNNYHMKTL LLYECEKHPR ETDWDEACLG DRLNGILLQL ISCLQCRRCP HYFLPNLDLF QGKPHSALES AAKQTWRLAR EILTNPKSLD KL
M. schreibersii    .......... .......... .......... .......... .......... .......... .......... .......... .......... .......... .......... .......... .......... .......... .......... .......... ..
M.lucifugus        .......... .......... .......... .......... .......... .......... .......... .......... .......... .......... .......... .......... .......... .......... .......... .......... ..
O.orca             .......... .......... .......... .......... .......... .......... .......... .......... .......... .......... .......... .......... .......... .......... .......... .......... ..
S.scrofa           .......... .......... .......... .......... .......... .......... .......... .......... .......... .......... .......... .......... .......... .......... .......... .......... ..
E.caballus         .......... .......... .......... .......... .......... .......... .......... .......... .......... .......... .......... .......... .......... .......... .......... .......... ..
C.hircus           .......... .......... .......... .......... .......... .......... .......... .......... .......... .......... .......... .......... .......... .......... .......... .......... ..
B.taurus           .......... .......... .......... .......... .......... .......... .......... .......... .......... .......... .......... .......... .......... .......... .......... .......... ..
C.lupus familiaris .......... .......... .......... .......... .......... .......... .......... .......... .......... .......... .......... .......... .......... .......... .......... .......... ..
H.sapiens          .......... .......... .......... .......... .......... .......... .......... .......... .......... ......S... .......... .......... .......... .......... .......... .......... ..
P.troglodytes      .......... .......... .......... .......... .......... .......... .......... .......... .......... ......S... .......... .......... .......... .......... .......... .......... ..
M.mulatta          .......... .......... .......... .......... .......... .......... .......... .......... .......... ......S... .......... .......... .......... .......... .......... .......... ..
P.anubis           .......... .......... .......... .......... .......... .......... .......... .......... .......... ......S... .......... .......... .......... .......... .......... .......... ..
S.boliviensis      .......... .......... .......... .......... .......... .......... .......... .......... .......... ......S... .......... .......... .......... .......... .......... .......... ..
O.garnettii        .......... .......... .......... .......... .......... .......... .......... .......... .......... ......S... .......... .......... .......... .......... .......... .......... ..
R.norvegicus       .......... .......... .......... .......... .......... .......... .......... .......... .......... .......... .......... .......... .......... .......... .......... .......... ..
M.ochrogaster      .......... .......... .......... .......... .......... .......... .......... .......... .......... .......... .......... .......... .......... .......... .......... .......... ..
H.glaber           .......... .......... .......... .......... .......... .......... .......... .......... .......... .......... .......... .......... .......... .......... .......... .......... ..
C.porcellus        .......... .......... .......... .......... .......... .......... .......... .......... .......... .......... .......... .......... .......... .......... .......... .......... ..
S.harrisii         .......... .......... .......... .......... .......... .......... .......... .......... .......... .......... .......... .......... .......... .......... .......... .......... ..
O.princeps         .......... .......... .......... .......... .......... .......... .......... .......... .......... .......... .......... .......... .......... .......... .......... .......... ..
```
