# Supplementary material for: Differential Expression of Meis2, Mab21l2 and Tbx3 during Limb Development Associated with Diversification of Limb Morphology in Mammals
Source: PLoS One. 2014 Aug 28;9(8):e106100. doi: 10.1371/journal.pone.0106100 (PMC4148388; doi:10.1371/journal.pone.0106100)

Figure S3. Alignment of amino acid sequences of *Tbx3* and species topologies of mammals. (A) Alignment of amino acid sequences of *Tbx3* in mammals. The protein domains of *Tbx3* were referred to the prediction of mouse *Tbx3* from Universal Protein Resource (http://www.uniprot.org/uniprot/P70324). (B) Species topologies of mammals used in the molecular evolutionary analysis of *Tbx3*.

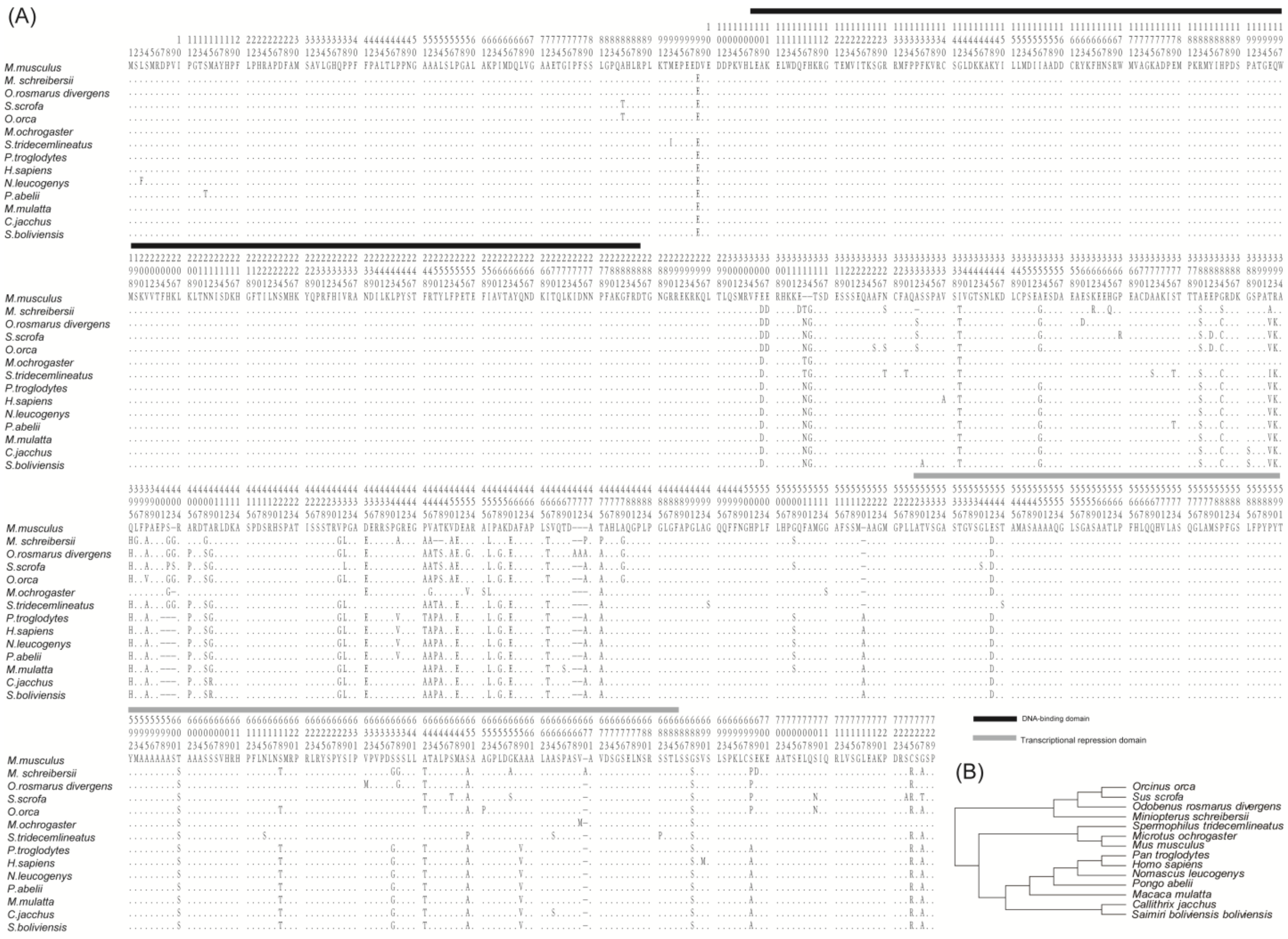

Supplement: Figure S3 — Alignment of amino acid sequences of Tbx3 and species topologies of mammals. (A) Alignment of amino acid sequences of Tbx3 in mammals. The protein domains of Tbx3 were referred to the prediction of mouse Tbx3 from Universal Protein Resource (http://www.uniprot.org/uniprot/P70324). (B) Species topologies of mammals used in the molecular evolutionary analysis of Tbx3. (PDF) [file pone.0106100.s003.pdf]
