# Supplementary material for: Differential Expression of Meis2, Mab21l2 and Tbx3 during Limb Development Associated with Diversification of Limb Morphology in Mammals
Source: PLoS One. 2014 Aug 28;9(8):e106100. doi: 10.1371/journal.pone.0106100 (PMC4148388; doi:10.1371/journal.pone.0106100)
Supplement: Table S1 — Primers for making WISH probes and for amplifying complete CDS of Bat Meis2 , Mab21l2 and Tbx3 . (PDF) [file pone.0106100.s004.pdf]

Table S1. Primers for making WISH probes and for amplifying complete CDS of Bat *Meis2*, *Mab21l2* and *Tbx3*.

**Gene**	**Forward primer (5’ → 3’)**	**Reverse primer (5’ → 3’)**	**Product length (bp)**
WISH probes			
Bat_Meis2	CACCCGTTGTTTCCTCTGTTAG	TCTCCACTCTGGGAAGCA	491
Mouse_Meis	TTACGGCGGGATGGACG	GGCGAGATTTGTGGAGGA	604
Bat_Mab21l2	GCCAGCCGCTCAATAACT'	CCTTGGGTTTCATTTTGTGGTG	550
Mouse_Mab21l2	CAGCCGCTCAATAACTACCA	GACATTGGCACTGAGACAC	744
Bat_TBx3	*TCCTCAACCTGAACACCAT*	AGATCCCGGATACAGAAACC	609
Mouse_TBx3	*GAAGTCAGGAAGGCGAATG*	TGGGCAAAGCAGTTGAAGG	543
CDS			
Bat_Meis2_CDS	ACTCTTCAGCCTAGATCACTT	GGCTTTGCGACTGCTTTA	1326
Bat_Mab21l2_CDS	CAGTGCGTGAGCCTTGGAT	CGTTCCGTTACGTCGCTTT	1188
Bat_Tbx3_CDS	CCAACAAACCAAAACAGCA	GGTGCCAACAGTGGAGACA	1089
(167 bp overlap)	CTGGAAACGGCAGGAGA	TGAGTTCCGAGCCCGAGT	1161
