# Supplementary material for: Differential Expression of Meis2, Mab21l2 and Tbx3 during Limb Development Associated with Diversification of Limb Morphology in Mammals
Source: PLoS One. 2014 Aug 28;9(8):e106100. doi: 10.1371/journal.pone.0106100 (PMC4148388; doi:10.1371/journal.pone.0106100)
Supplement: Table S2 — Information on species examined in amino acid alignment and molecular evolutionary analysis. (PDF) [file pone.0106100.s005.pdf]

Table S2. Information on species examined in amino acid alignment and molecular evolutionary analysis.

**Gene Name**	**Species Name**	**Accession *No.***
Meis2	*Homo sapiens*	NM_170674.4
	Pan troglodytes	XM_001137088.2
	Papio anubis	XM_003900742.1
	Nomascus leucogenys	XM_003272806.2
	Callithrix jacchus	XM_002753564.2
	Microtus ochrogaster	XM_005364206.1
	Mus musculus	XM_005381187.1
	Heterocephalus glaber	XM_004861520.1
	Ochotona princeps	XM_004578191.1
	Cavia porcellus	XM_003475668.2
	Ictidomys tridecemlineatus	XM_005316380.1
	Orcinus orca	XM_004274022.1
	Odobenus rosmarus divergens	XM_004409641.1
	Ceratotherium simum	XM_004421392.1
	Equus caballus	XM_005603163.1
	Bos mutus	XM_005904115.1
	Pantholops hodgsonii	XM_005968156.1
	Canis lupus familiaris	XM_005638216.1
	Myotis lucifugus	XM_006087500.1
	Miniopterus schreibersii	KJ670370
Mab21l2	*Homo sapiens*	NM_006439.4
	Pan troglodytes	XM_001151747.1
	Macaca mulatta	XM_001082398.2
	Papio anubis	XM_003899260.1
	Saimiri boliviensis	XM_003927983.1
	Otolemur garnettii	XM_003790215.1
	Rattus_norvegicus	NM_001109391.1
	Microtus ochrogaster	XM_005344172.1
	Mus musculus	NM_011839.3
	Heterocephalus glaber	XM_004869167.1
	Cavia porcellus	XM_003476901.1
	Sarcophilus harrisii	XM_003773139.1
	Ochotona princeps	XM_004588123.1
	Orcinus orca	XM_004263628.1
	Bos mutus	XM_005902365.1
	Capra hircus	XM_005691180.1
	Sus scrofa	XM_003129150.2
	Equus caballus	XM_001501565.3
	Canis lupus familiaris	XM_862437.3
	Myotis lucifugus	XM_006081771.1

	Miniopterus schreibersii	KJ670371
Tbx3	*Homo sapiens*	NM_005996.3
	Pan troglodytes	XM_001154479.2
	Nomascus leucogenys	XM_003274432.2
	Pongo abelii	XM_002823799.1
	Macaca mulatta	XM_001111958.1
	Callithrix jacchus	XM_002753045.1
	Saimiri boliviensis	XM_003932213.1
	Mus musculus	NM_198052.2
	Microtus ochrogaster	XM_005344349.1
	Spermophilus tridecemlineatus	XM_005329640.1
	Sus scrofa	XM_001927997.3
	Orcinus orca	XM_004281469.1
	Miniopterus schreibersii	KJ670372
	Odobenus rosmarus divergens	XM_004416807.1
