# Supplementary material for: Differential Expression of Meis2, Mab21l2 and Tbx3 during Limb Development Associated with Diversification of Limb Morphology in Mammals
Source: PLoS One. 2014 Aug 28;9(8):e106100. doi: 10.1371/journal.pone.0106100 (PMC4148388; doi:10.1371/journal.pone.0106100)
Supplement: Table S3 — Tajima relative rate tests of Tbx3 in mammls. (PDF) [file pone.0106100.s006.pdf]

Table S3. Tajima relative rate tests of *Tbx3* in mammls**.**

**Gene**	**Outgroup (C)**	**Testing group (A)**	**(B)**	**Mi**	**Md**	**MA**	**MB**	χ^2	**P value**
Tbx3	*H.sapiens*	*M. schreibersii*	*O. rosmarus divergens*	685	1	17	7	**4.17**	**<0.05**
			M. musculus	666	7	11	25	**5.44**	**<0.05**
			S. scrofa	681	2	17	11	1.29	>0.05
			O. orca	684	1	17	8	3.24	>0.05
			M. mulatta	691	1	23	1	**20.17**	**<0.05**
	S. scrofa	*M. schreibersii*	*H.sapiens*	681	2	17	8	3.24	>0.05
			M. musculus	663	7	11	26	**6.08**	**<0.05**
			O. rosmarus divergens	686	2	19	4	**9.78**	**<0.05**
			M. mulatta	683	1	18	6	**6.00**	**<0.05**
			O. orca	686	1	21	4	**11.56**	**<0.05**
	M. musculus	*M. schreibersii*	*H.sapiens*	666	7	11	7	0.89	>0.05
			O. rosmarus divergens	667	4	12	7	1.32	>0.05
			M. mulatta	667	5	12	6	2.00	>0.05
			S. scrofa	663	7	11	11	0.00	>0.05
			O. orca	665	5	10	9	0.05	>0.05

The Tajima relative rate test was used to examine the equality of evolutionary rate between bat (*M. schreibersii*) and other representative species with different outgorups.

Mi is the identical sites in all three sequences

Md is the divergent sites in all three sequences

MA is the number of unique differences in the sequence A

MB is the number of unique differences in the sequence B

χ^2 test statistic more than 3.841 indicates accelerated evolution

P value less than 0.05 is often used to reject the null hypothesis of equal rates between lineages
