# Supplementary material for: Differential Expression of Meis2, Mab21l2 and Tbx3 during Limb Development Associated with Diversification of Limb Morphology in Mammals
Source: PLoS One. 2014 Aug 28;9(8):e106100. doi: 10.1371/journal.pone.0106100 (PMC4148388; doi:10.1371/journal.pone.0106100)
Supplement: Table S4 — Branch model tests of selection pressure on Tbx3 gene in bats and rodent. (PDF) [file pone.0106100.s007.pdf]

Table S4. Branch model tests of selection pressure on *Tbx3* gene in bats and rodent.

Gene Name	Model	np	ℓ	ω_0	ω_b	ω_r	Model Compared	LRT	*P*-Value
Tbx3 (14 Species)	A. Free ratio Variable ω by branch	51	-7147.58	(0.0001-0.0830)	0.0440	0.0531			
	B. One ratio: $\omega_0 = \omega_b$	27	-7154.78	0.0306	$= \omega_0$	$= \omega_0$	B vs. A	14.4	0.9370
	C. Two ratios: ω_0, ω_b	28	-7153.63	0.0287	0.0439	$= \omega_0$	C vs. B	2.30	0.1294
	D. Two ratios: ω_0, ω_r	28	-7154.20	0.0624	$= \omega_0$	0.0299	D vs. B	1.16	0.2814
	E. Three ratio: ω_0, ω_b, ω_r	29	-7152.88	0.0278	0.0438	0.0643	E vs. C	1.50	0.2207
							E vs. D	2.64	0.1042

np, number of parameters.

Parameters ω_b, ω_r and ω_0 are the ω ratios for branches of the common ancestors of bats (ω_b) and rodents (ω_r) and the background (ω_0).
